# Nucleic acid sensor STING drives remodeling and its inhibition enhances steroid responsiveness in chronic obstructive pulmonary disease

**DOI:** 10.1371/journal.pone.0284061

**Published:** 2023-07-05

**Authors:** Bushra Mdkhana, Narjes Saheb Sharif-Askari, Rakhee K. Ramakrishnan, Baraa Khalid Al-Sheakly, Shirin Hafezi, Fatemeh Saheb Sharif-Askari, Khuloud Bajbouj, Qutayba Hamid, Rabih Halwani

**Affiliations:** 1 Research Institute for Medical and Health Sciences, University of Sharjah, Sharjah, United Arab Emirates; 2 Department of Family and Community Medicine and Behavioural Sciences, College of Medicine, University of Sharjah, Sharjah, United Arab Emirates; 3 Department of Pharmacy Practice and Pharmacotherapeutics, College of Pharmacy, University of Sharjah, Sharjah, United Arab Emirates; 4 Department of Basic Sciences, College of Medicine, University of Sharjah, Sharjah, United Arab Emirates; 5 Department of Clinical Sciences, College of Medicine, University of Sharjah, Sharjah, United Arab Emirates; 6 Meakins-Christie Laboratories, Research Institute of the McGill University Health Center, Montreal, Quebec, Canada; 7 Prince Abdullah Ben Khaled Celiac Disease Chair, Department of Pediatrics, Faculty of Medicine, King Saud University, Riyadh, Saudi Arabia; Chung Shan Medical University, TAIWAN

## Abstract

**Background:**

Chronic obstructive pulmonary disease (COPD) is progressive and irreversible chronic lung inflammatory disease. Cigarette smoke, the main cause of COPD, is often associated with double-stranded DNA release which potentially activates DNA-sensing pathways, such as STING. This study, therefore, analyzed the role of STING pathway in inducing pulmonary inflammation, steroid resistance, and remodeling in COPD.

**Methods:**

Primary cultured lung fibroblasts were isolated from healthy non-smoker, healthy smoker, and smoker COPD individuals. The expression of STING pathway, remodeling, and steroid resistance signatures were investigated in these fibroblasts upon LPS stimulation and treatment with dexamethasone and/or STING inhibitor, at both mRNA and protein levels using qRT-PCR, western blot, and ELISA.

**Results:**

At baseline, STING was elevated in healthy smoker fibroblasts and to a higher extent in smoker COPD fibroblasts when compared to healthy non-smoker fibroblasts. Upon using dexamethasone as monotherapy, STING activity was significantly inhibited in healthy non-smoker fibroblasts but showed resistance in COPD fibroblasts. Treating both healthy and COPD fibroblasts with STING inhibitor in combination with dexamethasone additively inhibited STING pathway in both groups. Moreover, STING stimulation triggered a significant increase in remodeling markers and a reduction in HDAC2 expression. Interestingly, treating COPD fibroblasts with the combination of STING inhibitor and dexamethasone alleviated remodeling and reversed steroid hyporesponsiveness through an upregulation of HDAC2.

**Conclusion:**

These findings support that STING pathway plays an important role in COPD pathogenesis, via inducing pulmonary inflammation, steroid resistance, and remodeling. This raises the possibility of using STING inhibitor as a potential therapeutic adjuvant in combination with common steroid treatment.

## Introduction

Chronic obstructive pulmonary disease (COPD) has been recently characterized by chronic airflow limitation caused by small airways and parenchymal destruction [1]. COPD is the third leading cause of death worldwide [2], and the largest contributor to chronic respiratory diseases burden [3]. The main characteristic features of this disease are the structural changes, remodeling of lung matrix and parenchymal destruction that caused by chronic inflammation [1]. Cigarette smoke is a main etiology of COPD, triggering airway inflammation and matrix remodeling which results in airway remodeling and steroid hyporesponsiveness [1, 4].

Pulmonary inflammation observed in smoking patients is mainly driven by oxidative DNA damage and subsequent cell death [5]. Smoker COPD patients have elevated levels of oxidative DNA damage in their peripheral blood, which in fact was proposed to identify patients at high mortality risk [6, 7]. Indeed, the cell death induced by cigarette smoke is mainly associated with the release of danger associated molecular patterns (DAMPs) as double-stranded (ds) DNA, which strongly correlated with neutrophilic airway inflammation, a characteristic of COPD [8, 9]. The cigarette smoke exposure induces the release of self-dsDNA in the bronchoalveolar space upon respiratory barrier damage [10], and nuclear and mitochondrial DNA damage in human endothelial cells [11]. However, the contribution of this mechanism to COPD pathogenesis as well as steroid resistance is not very well understood.

Cyclic GMP-AMP synthase (cGAS) and stimulator of interferon gene (STING) pathway represents the main dsDNA sensing pathway [12]. In the last decade, STING was identified as an endoplasmic reticulum (ER)-associated membrane protein, an adaptor for nucleic acid sensors, and intersected several intracellular signaling pathways. Detection of cytosolic ds-DNA by cGAS leads to the production of the second messenger cyclic GMP-AMP (cGAMP). cGAMP then binds to STING on ER resulting in its translocation to Golgi apparatus where TANK-binding kinase 1 (TBK1) is activated by autophosphorylation. Activated TBK1 in turn activates the transcriptional factors, interferon regulatory factor 3 (IRF3) and nuclear factor kappa-light-chain-enhancer of activated B cells (NF-κB), via phosphorylation, which translocates to the nucleus to induce transcription of type I interferon (IFN-I), and other inflammatory genes, including IL-6, respectively [12]. The release of self-dsDNA following cigarette smoke exposure activates the DNA sensor cGAS-STING pathway triggering IFN I-dependent lung inflammation [10]. Further, the cytosolic accumulation of nuclear and mitochondrial DNA in human endothelial cells following cigarette smoke exposure resulted activates the cGAS-STING pathway triggering IL-6 expression [11]. In addition, activated STING induced both pulmonary inflammation and fibrosis in a bleomycin mouse model [13]. Thus, the activation of STING pathway could contribute to COPD pathogenesis.

A key feature of COPD is the remodeling of lung matrix [14]. Fibroblasts, besides being an effector cells for fibrosis and resulting in the remodeling of the lung, maintain inflammation through the production of cytokines, chemokines, proteases, and lipid mediators [15]. Another major hallmark of COPD is hyporesponsiveness to corticosteroid treatment [16]. Interestingly, the innate immune responses, specifically through the cytosolic DNA sensing pathway, are differentially regulated by glucocorticoids [17]. Indeed, the STING-fibroblast-COPD axis and the role of STING in COPD as well as the effects of steroids on STING has not yet been explored. Therefore, in this study, using fibroblasts from smoker COPD patients and healthy subjects, we investigated the activation status of STING/IFN-I pathway and the rationale behind using pharmacological inhibition of STING as a potential adjuvant to enhance the anti-inflammatory effects of commonly used corticosteroids.

## Methodology

### Fibroblast cell culture

Human primary bronchial fibroblasts were isolated from endobronchial tissue biopsies and obtained from the Quebec Respiratory Health Research Network Tissue Bank (McGill University Health Centre (MUHC)/ Meakins-Christie Laboratories Tissue Bank, Montreal, Canada), as described previously [18, 19]. The original study was approved by the MUHC Research Ethics Board (2003–1879). The patient characteristics are summarized in **Table 1**. Non-asthmatic non-COPD healthy controls who are non-smokers and smokers, as well as smoker COPD patients were enrolled in the study [19]. The cells were cultured in Dulbecco’s modified Eagle’s medium (DMEM) (Sigma-Aldrich, Germany) supplemented with 10% fetal bovine serum (FBS), and 1% penicillin/streptomycin (Sigma-Aldrich, Germany). All incubations and cell maintenance were done in a humidified incubator at 37°C in 5% CO_2_. All the experiments were conducted at matched passages and a maximum of eight times.

**Table 1:** Demographic characteristics, and spirometry values of the enrolled subjects.

	Healthy Non-smoker	Healthy Smoker	Smoker COPD
	n = 4	n = 3	n = 4
**Age, years**	47.3 ± 12.5	57.0 ± 18.0	67.75 ± 6.9
**Male Gender**	2	2	2
**Caucasian Ethnicity**	4	3	4
**FEV1/FVC %**	-	-	56.7 ± 9.4
**Predicted FEV1%**	-	-	61.5% ± 10.6
**Current/Former Smoker**	-	1/2	2/2

Data are presented as mean ± SD

### Cigarette smoke extract (CSE) preparation

Marlboro Red cigarettes (Philip Morris, Victoria, Australia) has been used; each cigarette contained 1.1 mg of nicotine, 15 mg of tar, and 15 mg of carbon monoxide [20]. CSE was prepared using a pump-assisted bubbling method and smoked to 0.5 cm above the filter [21, 22]. One Marlboro cigarette was bubbled through 5 mL of PBS at a rate of 1 cigarette/min and this solution was considered as 100% concentration CSE. The 100% CSE was diluted to the appropriate concentration in culture media. Experiments were conducted with fibroblasts cultured at the same time utilizing the same CSE preparation to minimize experimental variability.

### Cell treatment

Fibroblasts from non-smoker healthy, smoker healthy and smoker COPD patients were seeded in 6- or 12-well-plates for experiments. When the cells reached ~70% confluency, they were serum-starved in 1% FBS supplemented DMEM for 16 hours (h). Then, the cells were stimulated with 5% of CSE for 8 h, or 10 μg/ml 2′,3′-cyclic guanosine monophosphate–adenosine monophosphate (2′,3′-cyclic GMP-AMP, cGAMP) (invivogen, USA) for 8 h, or with 10 μg/ml *pseudomonas aeruginosa* lipopolysaccharide (LPS) (Sigma-Aldrich, Germany) for 4 h, 8 h, or 24 h. For drug treatment, cells were stimulated with LPS (10 μg/ml) for 1 h then treated with dexamethasone (10nM) and/or STING antagonist, H151 (1μM), for the rest of the incubation period. LPS used as a model for COPD progression [23, 24].

### Quantitative Real Time-Polymerase Chain Reaction (qRT-PCR)

qRT-PCR assay was performed as previously described [25]. The total RNA was extracted using Trizol (Invitrogen) method according to manufacturer instructions. RNA concentrations and purity were measured using Nanodrop spectrophotometer (Thermo Scientific). cDNA synthesis was performed using High-Capacity cDNA Reverse Transcription Kit (Applied Biosystems) in the Veriti Thermal Cycler (Applied Biosystems). qRT-PCR reactions were carried out using 5x Hot FirePol EvaGreen qRT-PCR SuperMix (Solis Biodyne) in QuantStudio 3 Real-Time PCR System (Applied Biosystems). The primers are listed in **Table 2**. Gene expression was analyzed using the Comparative CT (ΔΔ CT) method after normalization to the housekeeping gene 18s rRNA. All results were expressed as mRNA expression (fold change) relative to healthy non-smoker for baseline measurements or unstimulated controls for treatment.

**Table 2:** List of primer sequences used in qRT-PCR.

Genes	Forward Primer Sequence (5ʹ-3ʹ)	Reverse Primer Sequence (5ʹ-3ʹ)
*STING*	GTACCTGGTGCTCCACCTAGCC	CCCGGTACCTGGAGTGGATGTG
*TBK1*	TTGCGAGATGTGGTGGGTGG	ACACAGACTGTCCATCTTCCCCT
*IFN-β*	CCTGTGGCAATTGAATGGGAGGC	AGATGGTCAATGCGGCGTCCTC
*IL-6*	GAAAGCAGCAAAGAGGCAC	GCACAGCTCTGGCTTGTTCC
*HDAC2*	CACCTGGTGTCCAGATGCAA	GCTATCCGCTTGTCTGATGCT
*TGF-β*	AAATTGAGGGCTTTCGCCTTA	GAACCCGTTGATGTCCACTTG
*COL1A1*	GATTGACCCCAACCAAGGCTG	GCCGAACCAGACATGCCTC
*COL3A1*	GATCAGGCCAGTGGAAATG	GTGTGTTTCGTGCAACCATC
*RelA*	GCCGAGTGAACCGAAACTCTGG	TTGTCGGTGCACATCAGCTTGC
*NF-κB1*	TCAGACGCCATCTATGACAGTAAAG	CTGGATGTCATCTTTCTGAACTTTG
*YAP1*	ACCCACAGCTCAGCATCTTC	GCTGTGACGTTCATCTGGGA
*18s*	CTACCACATCCAAGGAAGCA	TTTTTCGTCACTACCTCCCCG

### Western blot

Western blotting assay was performed as previously described [26]. The cells were lysed using RIPA lysis buffer (50mM Tris, 150mM NaCl, 1% sodium deoxycholate, 0.1% sodium-dodecyl-sulphate (SDS), 1% Triton X-100, pH7.5) after supplementation with 1x Protease Inhibitor Cocktail (Sigma-Aldrich, Germany) and 1 mM phenylmethylsulfonyl fluoride (Sigma-Aldrich, Germany). The protein lysates were quantified using ThermoScientific Pierce BCA Protein Assay Kit (ThermoFisher Scientific, US). Proteins were resolved in 8.5% or 12.5% SDS polyacrylamide gel, then transferred onto nitrocellulose membranes using semi-dry transfer cell. Membranes were incubated at 4°C with the following primary antibodies: anti-phospho-STING (#50907) (1:500), anti-STING (#13647) (1:1000), anti-phospho-TBK1/NAK (#5483) (1:500), anti-TBK1/NAK (#3504) (1:1000), anti-phospho-IRF3 (#29047) (1:500), anti-IRF3 (#11904) (1:1000), anti-COL1A1 (#66948) (1:1000), anti-MMP9 (#3852) (1:1000), anti-MMP2 (#40994) (1:1000), anti-MMP1 (#54376) (1:1000), and anti-β-actin (#4970) (1:1000) (Cell Signaling Technologies, Danvers, MA), anti-active YAP1 (#ab205270) (1:1000) (abcam) and anti-αSMA (#A5228) (1:1000) (Sigma-Aldrich, Germany). The membranes were then probed with horseradish peroxidase-conjugated secondary antibodies for 1 h. Sapphire™ NIR-Q Biomolecular Imager (Azure Biosystems, Dublin, US) was used to detect the protein bands, which were subsequently quantified using ImageJ software.

### Enzyme-linked immunosorbent assay (ELISA)

Cell culture supernatant was collected at 24 h of stimulation to determine the levels of IFN-β using standard ELISA kits according to the manufacturer’s instructions (Abcam).

### Statistical analysis

The results are presented as mean ± standard error of the mean (SEM) from four independent experiments *via* GraphPad Prism software, version 8.00 (GraphPad Software, Inc. La Jolla, CA, USA). Statistical comparisons were performed using unpaired independent student’s t-test for baseline expression comparison, and one-way ANOVA followed by the post hoc Bonferroni test for multiple comparisons. Statistical significance was accepted at a level of p < 0.05.

## Results

### Elevated STING activation in COPD fibroblasts and in response to cigarette smoke

**Fig 1:**
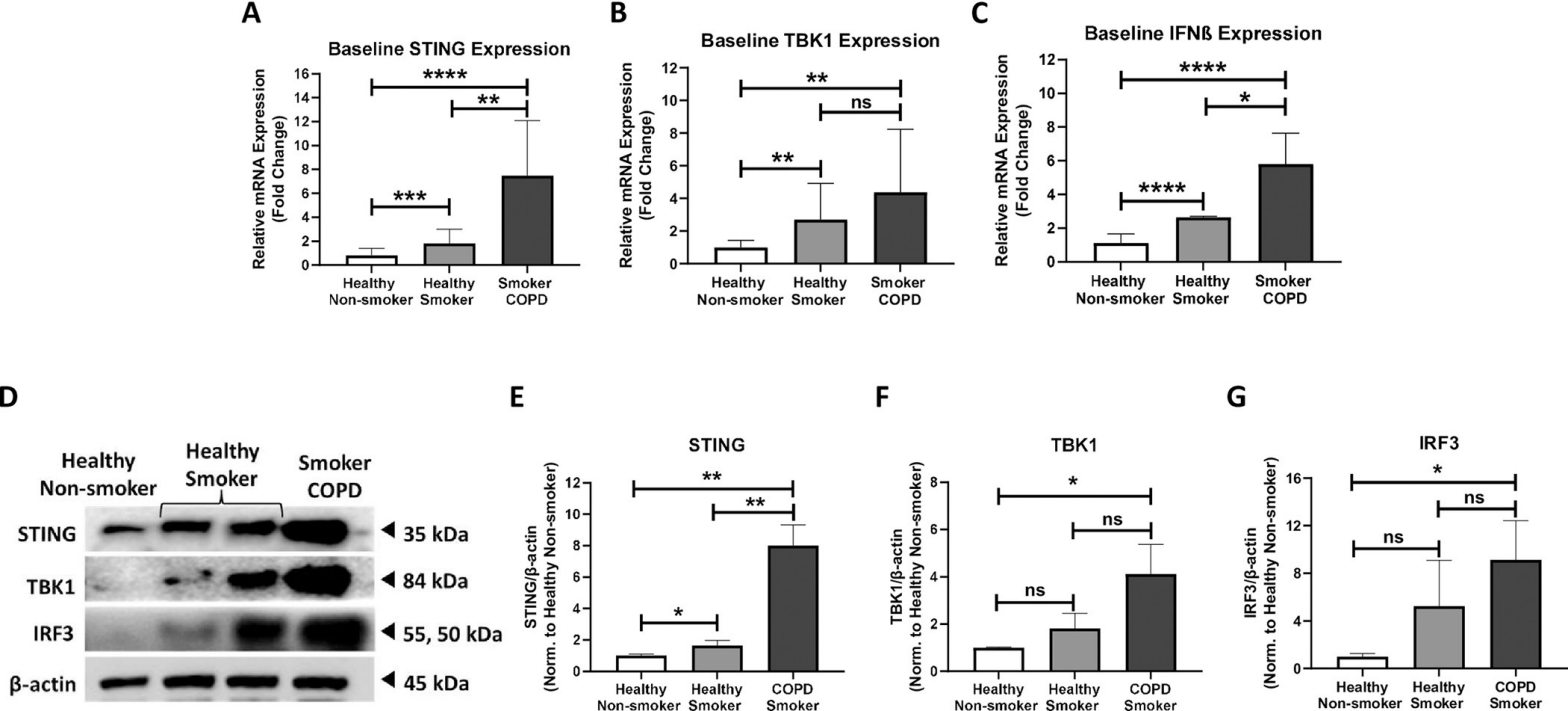
Elevated STING activation in COPD fibroblasts and in response to cigarette smoke. The baseline gene expression of STING **(A)**, TBK1 **(B)**, and IFN-β **(C)** in healthy non-smoker, healthy smoker, and smoker COPD fibroblasts. Data representative of 3–6 independent experiments from 3–4 unique donors in each group. Representative western blots of STING pathway in healthy non-smoker, healthy smoker, and smoker COPD fibroblasts **(D)**. Densitometric analysis of immunoblots depicting expression of STING **(E)**, TBK1 **(F)**, and IRF3 **(G)**. β-actin was used as a loading control. Data representative of 2–3 independent experiments from 2–3 unique donors in each group. Full blots are supplemented in **Fig A in S1 Raw images**. Results are presented as median (± interquartile ranges) and relative to healthy non-smoker fibroblasts. The values were compared across the different groups using one-way ANOVA followed by post hoc Bonferroni test for multiple comparisons. *p < 0.05, **p < 0.01, ***p < 0.001, ****p < 0.0001.

To examine the baseline profile of the STING pathway in COPD human lung fibroblasts, the mRNA and protein expression levels of intermediates in the STING pathway, such as STING, TBK1 and interferon type I (IFN-β), were examined in fibroblasts from healthy non-smokers, healthy smokers, and smoker COPD patients. At baseline, in comparison to healthy non-smoker fibroblasts, STING gene expression was higher in healthy smokers with a 2.2 fold increase (p<0.01) and in smoker COPD with a 6.9 fold increase (p<0.001) as shown in **Fig 1A**. The baseline expression of TBK1 gene was elevated in healthy smoker by 3.4 fold (p<0.01) and in smoker COPD by 4.5 fold (p<0.01) (**Fig 1B**). Similarly, IFN-β gene expression was increased in healthy smoker and to higher extent in smoker COPD, by 2.6 fold (p<0.001) and 5.8 fold (p<0.001), respectively (**Fig 1C**). At baseline, in comparison to healthy smoker, the gene expression of STING, TBK1, and IFN-β were also significantly higher in smoker COPD by 4.7 fold (p<0.01), 1.2 fold, and 3.2 fold (p<0.01), respectively (**Fig 1A–1C**).

We next investigated the protein expression of STING, TBK1, and IRF3 in these fibroblasts. STING protein level was elevated in healthy smoker by 1.7 fold (p<0.05), and in smoker COPD by 8.0 fold (p<0.01) when compared to healthy non-smoker fibroblasts (**Fig 1E**). Further, TBK1 protein level was higher in healthy smoker by 1.28 fold and in smoker COPD by 3.8 fold (p<0.05) (**Fig 1F**). In smoker COPD, the protein level of IRF3 was increased by 9.1 fold (p<0.05) and in healthy smoker by 5.2 fold in comparison to healthy non-smokers (**Fig 1G**). Similarly, STING, TBK1, and IRF3 protein levels were elevated in smoker COPD in comparison to healthy smoker fibroblasts by 6.3 fold (p<0.01), 2.0 fold, and 3.9 fold, respectively (**Fig 1E–1G**). These results suggest that STING pathway is activated in response to cigarette smoking and is elevated in COPD fibroblasts.

### STING pathway is suppressed by dexamethasone in healthy non-smoker fibroblasts and STING inhibitor induced an additive effect

**Fig 2:**
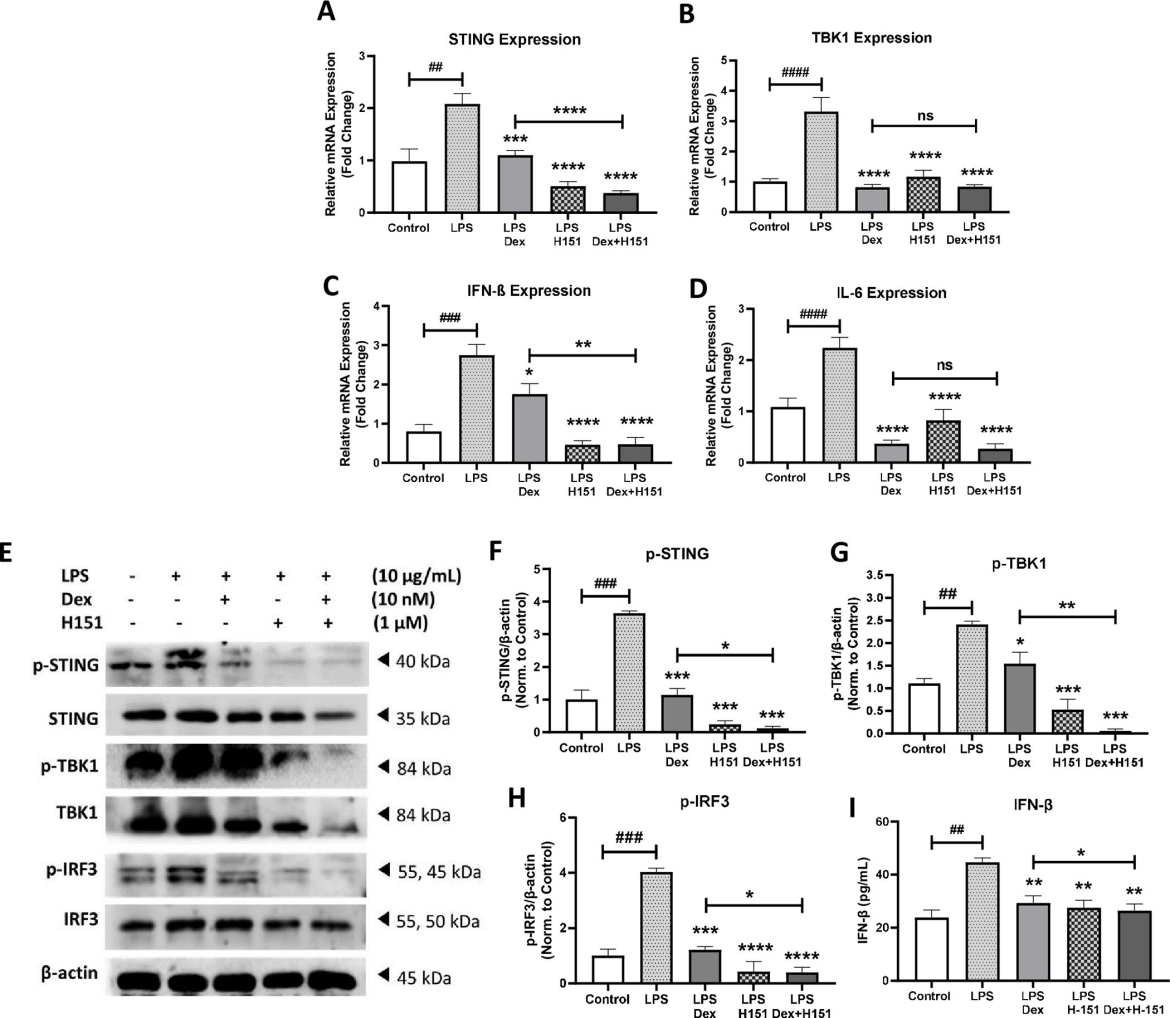
STING pathway is suppressed by dexamethasone in healthy non-smoker fibroblasts and STING inhibitor induced an additive effect. The gene expression of STING **(A)**, TBK1 **(B)**, IFN-β **(C),** and IL-6 **(D)** was compared upon stimulation with LPS for 4 h, then treatment with dexamethasone (Dex), and STING inhibitor (H151) either alone or in combination in healthy non-smoker fibroblasts. Data representative of 2–3 independent experiments from four unique donors. At indicated concentration of the stimulant and treatment for 8 h, representative western blots **(E)**, and densitometric analysis of immunoblots depicting the expression of STING pathway members **(F-H)**. Data representative from two unique donors. β-actin was used as a loading control. Full blots are supplemented in **Fig B in S1 Raw images**. Concentration of type I IFN (IFN-β) in the culture media upon 24 h stimulation was analyzed by ELISA **(I)**. Data representative from two unique donors. Results are presented as mean (± SEM) and relative to unstimulated control. The values were compared across the different groups using one-way ANOVA followed by post hoc Bonferroni test for multiple comparisons. *p < 0.05, **p < 0.01, ***p < 0.001, ****p < 0.0001 vs. LPS stimulation. ###p < 0.001, ####p < 0.0001 vs. unstimulated control.

Healthy non-smoker fibroblasts were first stimulated with LPS from *pseudomonas aeruginosa* as LPS is known to activate the STING pathway [27]. Upon LPS stimulation for 4 h, STING, TBK1, IFN-β, and IL-6 gene expression levels were upregulated by 2.3 fold (p<0.01), 3.3 fold (p<0.0001), 2.5 fold (p<0.001), and 2.8 fold (p<0.0001), respectively in comparison to unstimulated control, as shown in **Fig 2A–2D**. Upon treatment with dexamethasone, the upregulated STING, TBK1, IFN-β and IL-6 were significantly decreased by 1.0 fold (p<0.001), 0.8 fold (p<0.0001), 2.2 fold (p<0.05), and 0.3 fold (p<0.0001), respectively in comparison to LPS stimulation (**Fig 2A–2D**). We next proposed the use of dexamethasone in combination with STING inhibitor (H151); where H151 is a recently discovered potent, irreversible, and selective inhibitor of STING [28]. The mRNA levels of STING, and IFN-β were significantly downregulated by 0.6 fold (p<0.0001), and 1.4 fold (p<0.01), respectively, upon the combination treatment when compared to dexamethasone single treatment (**Fig 2A and 2C**).

The effect of the proposed treatment was also confirmed on a protein level by western blot analysis. The activation of STING, TBK1, and IRF3 by phosphorylation was induced upon LPS stimulation for 8 h by 3.6 fold (p<0.001), 2.4 fold (p<0.01), and 4.0 fold (p<0.001), respectively, relative to unstimulated control. The induced activity and expression of these proteins, STING, TBK1, and IRF3, were significantly inhibited when treated with dexamethasone in comparison to LPS stimulation by 1.1 fold (p<0.001), 1.5 fold (p<0.05), 1.2 fold (p<0.001), respectively. Then, the proposed dexamethasone and H151 combination inhibited the phosphorylation of STING, TBK1, and IRF3 by 1.0 fold (p<0.05), 1.5 fold (p<0.01), and 0.9 fold (p<0.05), respectively in comparison to dexamethasone treatment (**Fig 2E–2H**). The combination treatment further suppressed IFN-β secretion by 9.8% (p<0.05) after 24 h stimulation compared with dexamethasone treatment alone (**Fig 2I**). In overall, STING inhibitor H151 augmented the anti-inflammatory effect of dexamethasone.

### STING activity in COPD fibroblasts is resistant to dexamethasone monotherapy but responsive to combination therapy of STING inhibitor and dexamethasone

**Fig 3:**
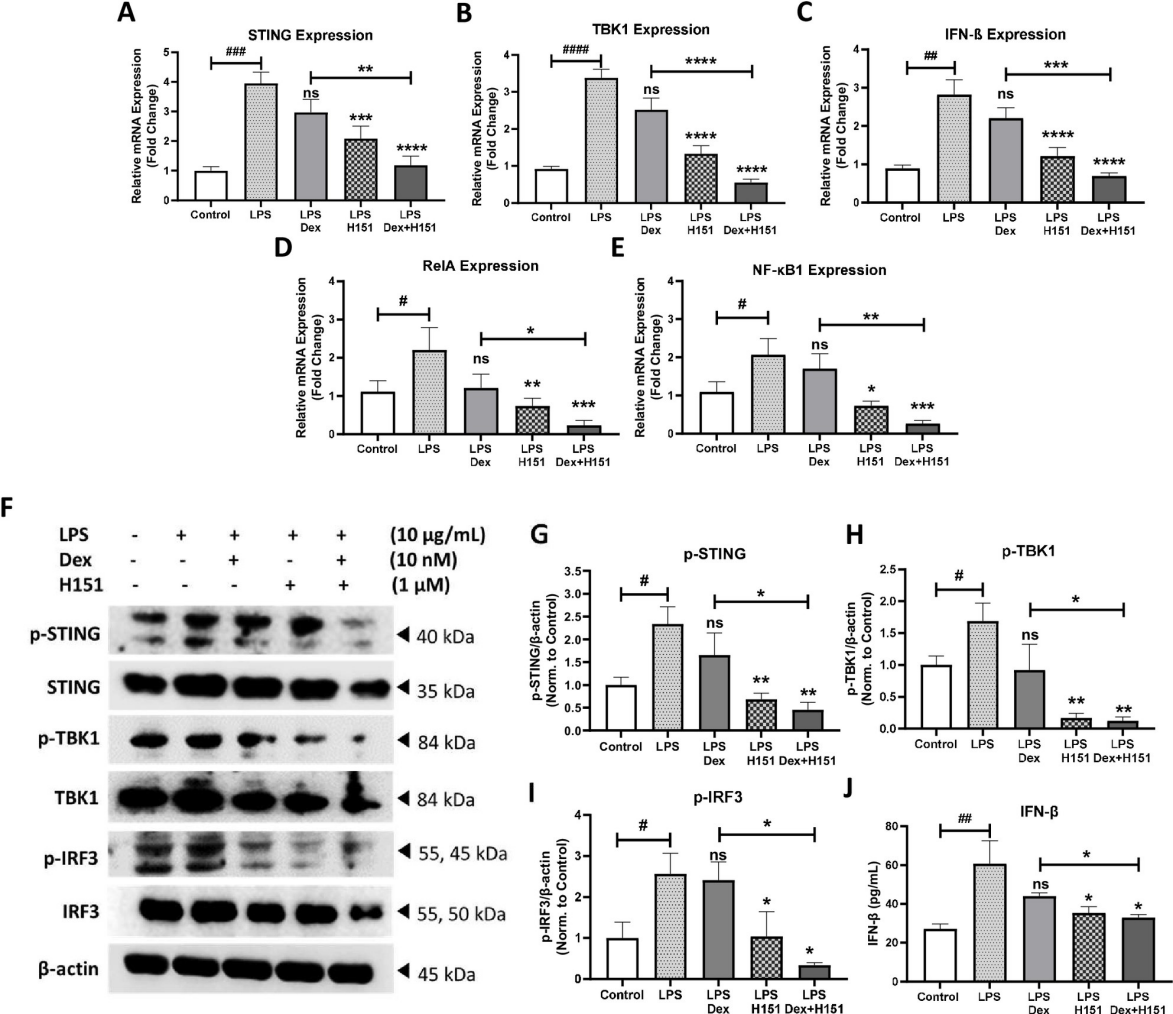
STING activity in COPD fibroblasts is resistant to dexamethasone monotherapy but responsive to combination therapy of STING inhibitor and dexamethasone. The gene expression of STING **(A)**, TBK1 **(B)**, IFN-β **(C)**, RelA **(D),** and NF-Κb1 **(E)** was compared upon stimulation for 4 h with LPS, then treatment with Dex, H151 and combination in smoker COPD fibroblasts. Data representative of 2–3 independent experiments from four unique donors. Representative western blots **(F)**, and densitometric analysis of STING pathway members in smoker COPD fibroblasts at indicated concentration of the stimulant and treatment for 8 h **(G-I)**. β-actin was used as a loading control. Data representative from three unique donors. Full blots are supplemented in **Fig C in S1 Raw images**. Concentration of IFN-β in the culture media after 24 h of stimulation was analyzed by ELISA **(J)**. Data representative from two unique donors. Results are presented as mean (± SEM) and relative to unstimulated control. The values were compared across the different groups using one-way ANOVA followed by post hoc Bonferroni test for multiple comparisons. *p < 0.05, **p < 0.01, ***p < 0.001, ****p < 0.0001 vs. LPS stimulation. ###p < 0.001, ####p < 0.0001 vs. unstimulated control.

Since STING pathway was activated in smoker COPD fibroblasts (**Fig 1**), we next investigated whether the commonly used steroid treatment, dexamethasone, inhibits STING pathway in in-vitro model of COPD exacerbation. First, smoker COPD fibroblasts were stimulated for 4 h with *pseudomonas aeruginosa* LPS, then treated with dexamethasone. The expression of STING, TBK1, and IFN-β genes were upregulated upon LPS stimulation with 4.1 fold (p<0.01), 3.4 fold (p<0.0001), and 3.7 fold (p<0.01), respectively in comparison to unstimulated control. However, upon dexamethasone treatment, the increased mRNA levels of STING, TBK1, and IFN-β specifically were not significantly reduced when compared to LPS stimulation. Interestingly, combination treatment of dexamethasone and H151 showed a significant reduction in the gene expression of STING, TBK1, and IFN-β by 1.8 fold (p<0.01), 2.0 fold (p<0.0001), and 1.5 fold (p<0.001), respectively, when compared to dexamethasone treatment as presented in **Fig 3A–3C**.

In parallel, STING oligomerization also shown to activate the IKK complex leading to NF-κB activation, in particular, RelA (p65) and NF-κB1 (p50) [29], which a critical inflammatory mediator in COPD [30]. Therefore, RelA and NF-κB1 at mRNA level was investigated upon combination treatment of dexamethasone and H151. The gene expression of RelA and NF-κB1 were not significantly changed upon treatment with dexamethasone in comparison to LPS stimulation. Importantly, a significant reduction in the mRNA level of RelA and NF-κB1 by 0.9 fold (p<0.05), and 1.4 fold (p<0.01), respectively, when compared to dexamethasone single treatment (**Fig 3D and 3E**).

In smoker COPD fibroblasts, both the expression on protein level and the activity were affected as per the proposed treatment plan. Upon LPS stimulation for 8 h, the phosphorylation of STING, TBK1, and IRF3 was increased by 1.9 fold (p<0.05), 1.7 fold (p<0.05), and 2.6 fold (p<0.05), respectively. Then, the expression of these proteins, STING, TBK1, and IRF3, were not significantly inhibited when treated with dexamethasone in comparison to LPS stimulation. The combination treatment of dexamethasone and H151 decreased the phosphorylation levels of STING, TBK1, and IRF3 when compared to dexamethasone treatment by 1.2 fold (p<0.05), 0.8 fold (p<0.05), and 1.2 fold (p<0.05), respectively (**Fig 3F–3I**). Further, we investigated the effect of combination treatment on LPS-induced type I IFN secretion. Combination treatment caused 25.3% (p<0.05) reduction of IFN-β level after 24 h stimulation compared with dexamethasone treatment (**Fig 3J**). Altogether, these results demonstrate that the inhibition of the STING pathway enhanced the steroid responsiveness of COPD fibroblasts.

### Inhibition of STING pathway augments steroid responsiveness in COPD fibroblasts by upregulating HDAC2 levels

**Fig 4:**
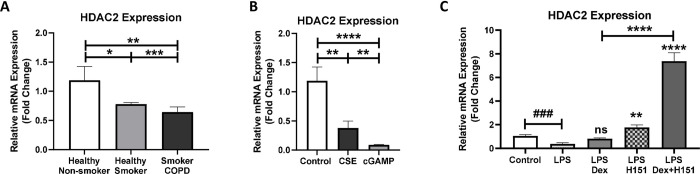
The combination of STING inhibitor and dexamethasone augments steroid responsiveness in COPD fibroblasts by upregulating HDAC2. **(A)** The baseline gene expression of HDAC2 in healthy non-smoker, healthy smoker, and smoker COPD fibroblasts. Data representative of 2–4 independent experiments from 2–3 unique donors in each group. **(B)** In healthy non-smoker fibroblasts, comparison of HDAC2 gene expression upon stimulation for 8 h with CSE, and 2’3’-cGAMP. Data representative of 2–3 independent experiments from two unique donors. **(C)** In smoker COPD fibroblasts, comparison of HDAC2 gene expression upon stimulation for 4 h with LPS, then treatment with Dex, H151 and combination. Data representative from four unique donors. Results are presented as mean (± SEM) and relative to unstimulated control. The values were compared across the different groups using one-way ANOVA followed by post hoc Bonferroni test for multiple comparisons. *p < 0.05, **p < 0.01, ***p < 0.001, ****p < 0.0001 vs. LPS stimulation. ###p < 0.001, ####p < 0.0001 vs. unstimulated control.

Corticosteroid treatment hyporesponsiveness is one of the key characteristic features of COPD; which is associated with decrease in activity and expression of histone deacetylase 2 (HDAC2) [16]. Here, the mRNA baseline expression levels of HDAC2 in smoker COPD human lung fibroblasts was examined in comparison to healthy non-smokers, and healthy smokers. HDAC2 gene expression was lower in healthy smokers by 0.8 fold (p<0.05) and to a lower extent in smoker COPD by 0.6 fold (p<0.01) as shown in **Fig 4A**. Healthy non-smoker fibroblasts stimulated with mammalian STING ligand, cGAMP (2’-3’-cyclic GMP-AMP), for 8 h inhibited HDAC2 gene expression by almost 1.0 fold (p<0.0001), when compared to unstimulated control. Interestingly, cGAMP stimulation significantly inhibited HDAC2 expression to higher extent (p<0.01) in comparison to CSE (**Fig 4B**). LPS stimulation in smoker COPD fibroblasts for 4 h reduced HDAC2 mRNA level by 0.4 fold (p<0.001), relative to unstimulated control. The expression of HDAC2 was not significantly induced upon treatment with dexamethasone when compared to LPS stimulation. However, the combination treatment of dexamethasone and STING inhibitor (H151) showed a significant induction in HDAC2 gene expression by 6.5 fold increase (p<0.0001), in comparison to dexamethasone single treatment (**Fig 4C**). This indicated that the proposed combination therapy may improve steroid responsiveness in COPD by enhancing HDAC2 expression.

### STING inhibitor in combination with dexamethasone alleviates remodeling in steroid resistant COPD fibroblasts

**Fig 5:**
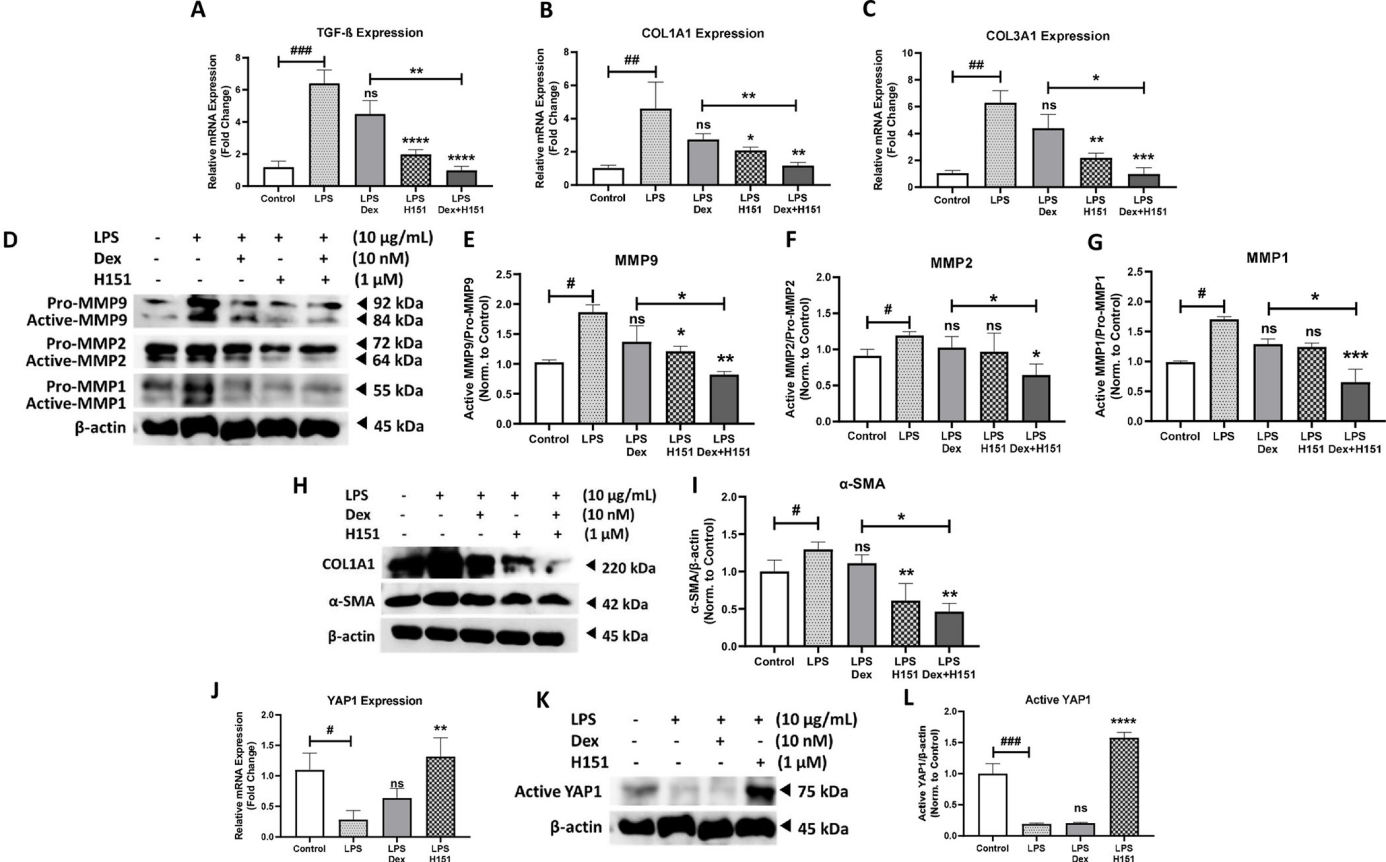
STING inhibitor in combination with dexamethasone alleviates remodeling in steroid resistant COPD fibroblasts. Comparison of COL1A1 **(A)**, COL3A1 **(B)**, and TGF-β **(C)** gene expression upon stimulation for 4 h with LPS, then treatment with Dex, H151 and combination in smoker COPD fibroblasts. Data representative of two independent experiments from four unique donors. Representative western blot analysis and densitometric analysis of remodeling mediators in smoker COPD fibroblasts at indicated concentration of the stimulant and treatment for 24 h **(D-I)**. β-actin was used as a loading control. Data representative from two independent experiments. Full blots are supplemented in **Fig D in S1 Raw images**. **(J)** The gene expression of YAP1 was compared upon stimulation for 4 h with LPS, then treatment with Dex, H151 and combination in smoker COPD fibroblasts. Data representative from four unique donors. Representative western blot **(K),** and densitometric analysis of active YAP1 in smoker COPD fibroblasts at indicated concentration of the stimulant and treatment for 8 h **(L).** β-actin was used as a loading control. Data representative from two independent experiments. Full blots are supplemented in **Fig D in S1 Raw images**. Results are presented as mean (± SEM) and relative to unstimulated control. The values were compared across the different groups using one-way ANOVA followed by the post hoc Bonferroni test for multiple comparisons. *p < 0.05, **p < 0.01, ***p < 0.001, ****p < 0.0001 vs. LPS stimulation. ###p < 0.001, ####p < 0.0001 vs. unstimulated control.

STING pathway plays an essential role in inducing pulmonary fibrosis and remodeling via stimulating the expression of remodeling hallmarks, such as collagen I (COL1A1), matrix metalloproteinase9 (MMP9), and TGF-β1 [13]. Further, our group recently published that LPS induced pro-fibrotic signalling in severe asthmatic bronchial fibroblasts [31]. Therefore, LPS was used as stimulated for fibrosis in smoker COPD fibroblasts. Here, stimulating smoker COPD fibroblasts with LPS for 4 h induced the gene expression of TGF-β1, COL1A1, and COL3A1 by 5.5 fold (p<0.0001), 4.9 fold (p<0.05), and 5.5 fold (p<0.01), respectively, relative to unstimulated control. Upon treatment with dexamethasone, the expression of these remodeling markers was not significantly reduced when compared to LPS stimulation. Treatment with dexamethasone in combination with STING inhibitor (H151) showed a significant reduction in the mRNA levels of TGF-β1, COL1A1, and COL3A by 2.7 fold (p<0.0001), 1.4 fold (p<0.01), and 2.5 fold (p<0.05), respectively, when compared to dexamethasone single treatment (**Fig 5A–5C**).

Interestingly, the active MMP9, MMP2, and MMP1 protein levels were increased upon LPS stimulation for 24 h by 1.9 fold (p<0.05), 1.2 fold (p<0.05), and 1.7 fold (p<0.05), respectively, in comparison to unstimulated control. The elevated active MMPs, MMP9, MMP2, and MMP1, were not significantly inhibited when treated with dexamethasone compared to LPS stimulation. Interestingly, the combination treatment decreased the active protein level of MMP9, MMP2, and MMP1 by 0.4 fold (p<0.05), 1.0 fold (p<0.05), and 0.4 fold (p<0.05), respectively, when compared to dexamethasone treatment alone (**Fig 5D–5G**). In parallel, the COL1A1 and α-SMA protein levels were increased upon LPS stimulation for 24 h by 2.8 fold and 1.3 fold (p<0.05), respectively, in comparison to unstimulated control. The elevated proteins, COL1A1 and α-SMA, were not significantly inhibited when treated with dexamethasone compared to LPS stimulation. Interestingly, the combination treatment decreased the protein level of COL1A1 and α-SMA by 0.3 fold, 0.6 fold (p<0.05), respectively, when compared to dexamethasone treatment (**Fig 5H and 5I**).

It has been reported that YAP1 deficiency is associated with lung emphysema [32], and that STING is involved in suppressing YAP1 [33, 34]. Therefore, we studied how YAP1 expression and activation is influenced by STING pathway suppression. YAP1 mRNA level was reduced (p<0.05) when stimulated smoker COPD fibroblasts with LPS for 4 h in comparison to unstimulated control. The expression of YAP1 was not significantly induced upon treatment with dexamethasone when compared to LPS stimulation. However, STING inhibitor (H151) treatment showed a significant induction by 1 fold increase (p<0.01) in YAP1 gene expression when compared to LPS stimulation (**Fig 5J**). Interestingly, active YAP1 protein level was further decreased (p<0.001) upon LPS stimulation for 24 h relative to control, which was not significantly induced upon dexamethasone treatment. Indeed, active YAP1 protein was significantly induced by 1.6 fold (p<0.0001) when treated with STING inhibitor (H151) compared to LPS stimulation (**Fig 5K and 5L**). Altogether these results indicate that STING inhibitor augmented the effect of steroids in inhibiting remodeling in COPD.

## Discussion

In this study, STING pathway was found to be upregulated in primary fibroblasts isolated from healthy smokers when compared to non-smokers, and to a higher extent in smokers with COPD. In smoker COPD patients, steroid treatment did not suppress these pathways, while a combination of steroid and STING inhibitor significantly reduced the expression of STING/IFN-β signaling and lowered the expression of remodeling markers. Furthermore, the combination of STING inhibitor and Dexamethasone restored steroid sensitivity through induction of HDAC2 gene expression. Overall, the activated STING pathway plays a key role in COPD pathogenesis through the induction of both IFN-I response and lung remodeling. STING inhibitor, H151, could be used as a potential adjuvant to steroid medication.

STING is a part of innate immune signaling pathway that senses cytosolic dsDNA and leads to type I IFN response [10, 35, 36]. This pathway is known to drive inflammation in different chronic diseases, such as systemic lupus erythematosus [37–39], rheumatoid arthritis [40], atherosclerosis [11], acute lung injury [27, 41, 42], silicosis [35, 43], and COPD [10, 44]. The respiratory barrier damage upon cigarette smoke exposure triggers cell-free DNA and oxidative DNA damage [5, 8, 10], that could be detected in the peripheral blood of COPD patients [6, 45]. Thus, the released self-dsDNA triggers DNA sensor cGAS-STING pathway, inducing IFN I-dependent lung inflammation [10]. In peripheral blood and bronchial brushings from COPD patients, the gene expression signature of IFN signaling, particularly, IFN-stimulated genes (ISGs), positively correlated with exacerbations, airway remodeling and impaired lung function [46, 47]. In addition, IFN-β and -λ were found to be higher in sputum from COPD patients compared to healthy volunteers, which positively correlated with sputum neutrophil count, one of the key features of COPD [48]. Further, using nontypeable *Haemophilus influenzae* as a model for COPD exacerbation, STING played an essential role in IFN-β expression [44]. Following the previous findings, we have shown substantial enrichment of the baseline STING profile in COPD fibroblasts (**Fig 1**), which was further elevated upon LPS stimulation at both mRNA and protein levels (**Fig 3**).

Glucocorticoids differentially target the innate immune responses through the cytosolic DNA sensing pathway [17]. In LPS stimulated primary macrophages, dexamethasone treatment suppressed TBK1 activation; inhibiting IRF3 phosphorylation [49], disrupted IRF3-glucocorticoid receptor protein complex [50], and decreased IFN-I secretion [17, 51]. Supporting these findings, here we showed STING/IFN-β pathway was responsive to dexamethasone in healthy non-smoker fibroblasts (**Fig 2**). However, dexamethasone did not affect the activity of this pathway in fibroblasts from COPD patients (**Fig 3**), while STING inhibitor had an additive anti-inflammatory effect to steroid upon combination of STING inhibitor and commonly used steroid treatment, dexamethasone (**Figs 2** and **3**). Indeed, we have detected the additive effect in COPD mRNA and protein levels (**Fig 3**). For contrast, it showed at protein level but not mRNA expression in healthy fibroblasts (**Fig 2**). The reason for that could be that mRNA changes occur at different time point in healthy compared to COPD.

Corticosteroids utilize histone deacetylases (HDACs) to suppress inflammatory gene expression [16]. In COPD, oxidative stress and smoking suppress HDAC2 expression and activity and result in steroid hyporesponsiveness [16]. Likewise, our results showed that HDAC2 expression was lower in COPD fibroblasts (**Fig 4A**). While, selective STING stimulant, 2’,3’-cGAMP, significantly reduced HDAC2 expression in healthy fibroblasts in comparison to CSE (**Fig 4B**), the combination of STING inhibitor and dexamethasone in COPD fibroblasts reversed the steroid hyporesponsiveness through upregulation of HDAC2 (**Fig 4C**). To the best of our knowledge, this is the first study to associate the HDAC2 attenuation to DNA damage and STING upregulation. The link between STING signaling and HDAC2 expression is not well investigated and requires further evaluation.

Recently, STING signaling was reported to induce fibrosis in cardiac [52], hepatic [53], renal [54], and pulmonary inflammation [13]; via the upregulation of fibrotic hallmarks, transforming growth factor beta (TGF-β), and collagen I (COL1A1) [13, 52–54]. From a mechanistic perspective, the binding of cGAMP to STING on endoplasmic reticulum (ER), directly activates the ER-located kinase phospho-protein kinase RNA-like endoplasmic reticulum kinase (PERK). The activated PERK phosphorylates phospho-eukaryotic initiation factor 2 alpha (eIF2α); this STING–PERK–eIF2α pathway is physiologically essential to senescence and fibrosis [55]. Further, genetic depletion of STING restrains ER stress, decreasing the activation of PERK–eIF2α pathway [52]. Therefore, STING could be a therapeutic target for lung fibrosis. Our data in the line with the previous findings, where STING stimulation in COPD fibroblasts triggered significant increase in remodeling markers, TGF-β, alpha-smooth muscle actin (α-SMA), COL1A1, collagen III (COL3A1), and MMPs, while treatment with STING inhibitor, H151, in combination with dexamethasone reduced them (**Fig 5A–5I**).

cGAS-STING signaling pathway was also associated with the suppression of YAP1 in endothelial cells [33] and non-small cell lung cancer [34]. Likewise, our results showed that treatment with STING inhibitor significantly induced YAP1 at both mRNA and protein levels (**Fig 5J–5L**). Age played a role in cGAS-STING induced endothelial dysfunction in cardiovascular diseases [56], however, its effect in respiratory diseases remains unknown. Age was not adjusted in our study which could or not affect the results.

The current work underscores the importance of STING pathway in COPD pathogenesis specifically in primary fibroblasts isolated from COPD patients; via inducing IFN-I secretion, pulmonary inflammation, and remodeling. Thus, STING inhibitor might represent a potential therapeutic adjuvant in combination with routine steroid treatment to enhance steroid sensitivity and to control lung inflammation and remodeling, thereby preventing COPD progression.

## Supporting information

S1 Raw images (PDF)

S1 File (PDF)
