# Supplementary material for: Nucleic acid sensor STING drives remodeling and its inhibition enhances steroid responsiveness in chronic obstructive pulmonary disease
Source: PLoS One. 2023 Jul 5;18(7):e0284061. doi: 10.1371/journal.pone.0284061 (PMC10321631; doi:10.1371/journal.pone.0284061)

Figure A:

Baseline Expression

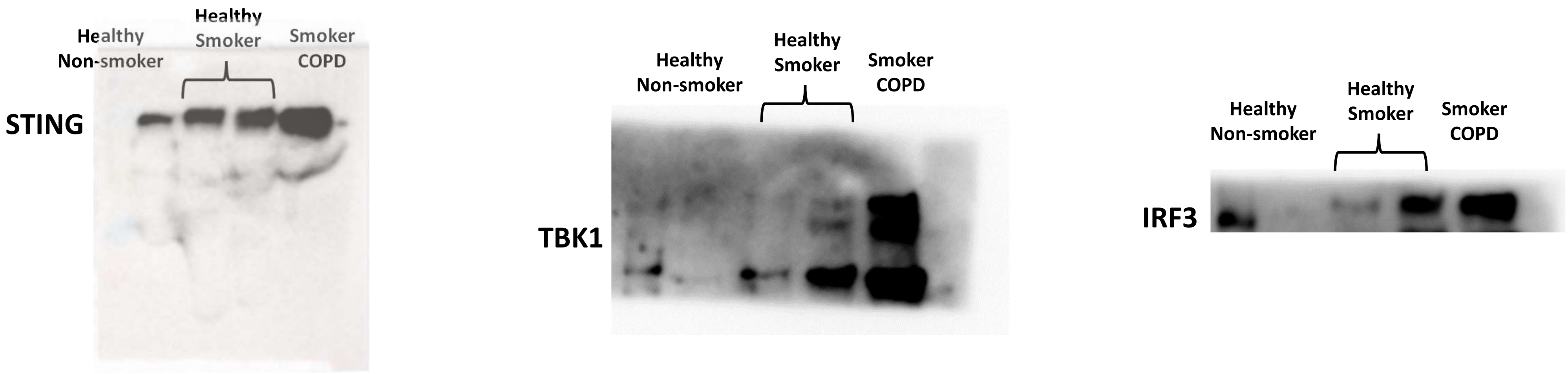

Figure B:

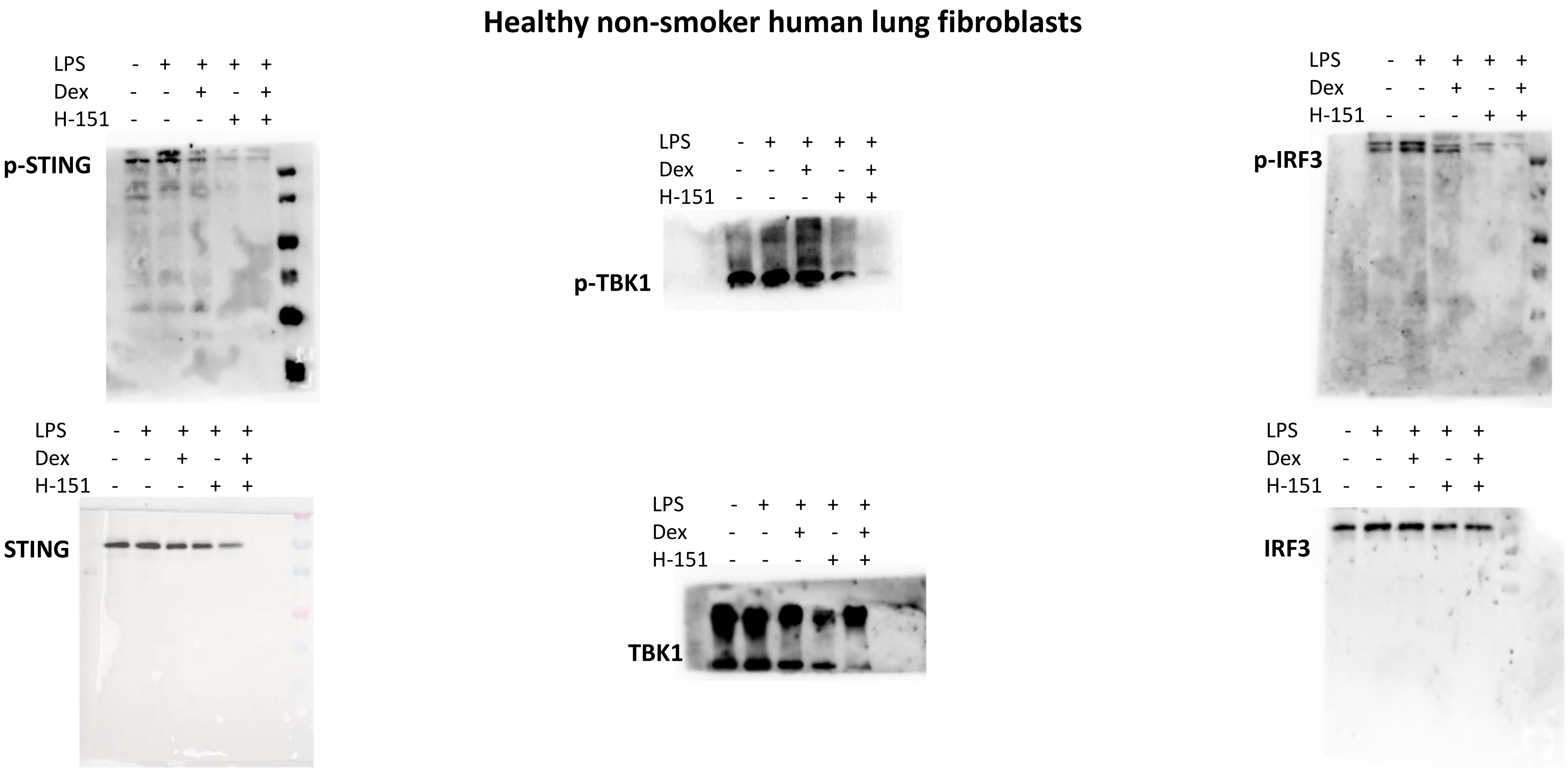

Healthy non-smoker human lung fibroblasts
LPS - + + + +
Dex - - + - +
H-151 - - - + +
p-STING
LPS - + + + +
Dex - - + - +
H-151 - - - + +
p-TBK1
LPS - + + + +
Dex - - + - +
H-151 - - - + +
p-IRF3
LPS - + + + +
Dex - - + - +
H-151 - - - + +
STING
LPS - + + + +
Dex - - + - +
H-151 - - - + +
TBK1
LPS - + + + +
Dex - - + - +
H-151 - - - + +
IRF3

Figure C:

Smoker COPD human lung fibroblasts

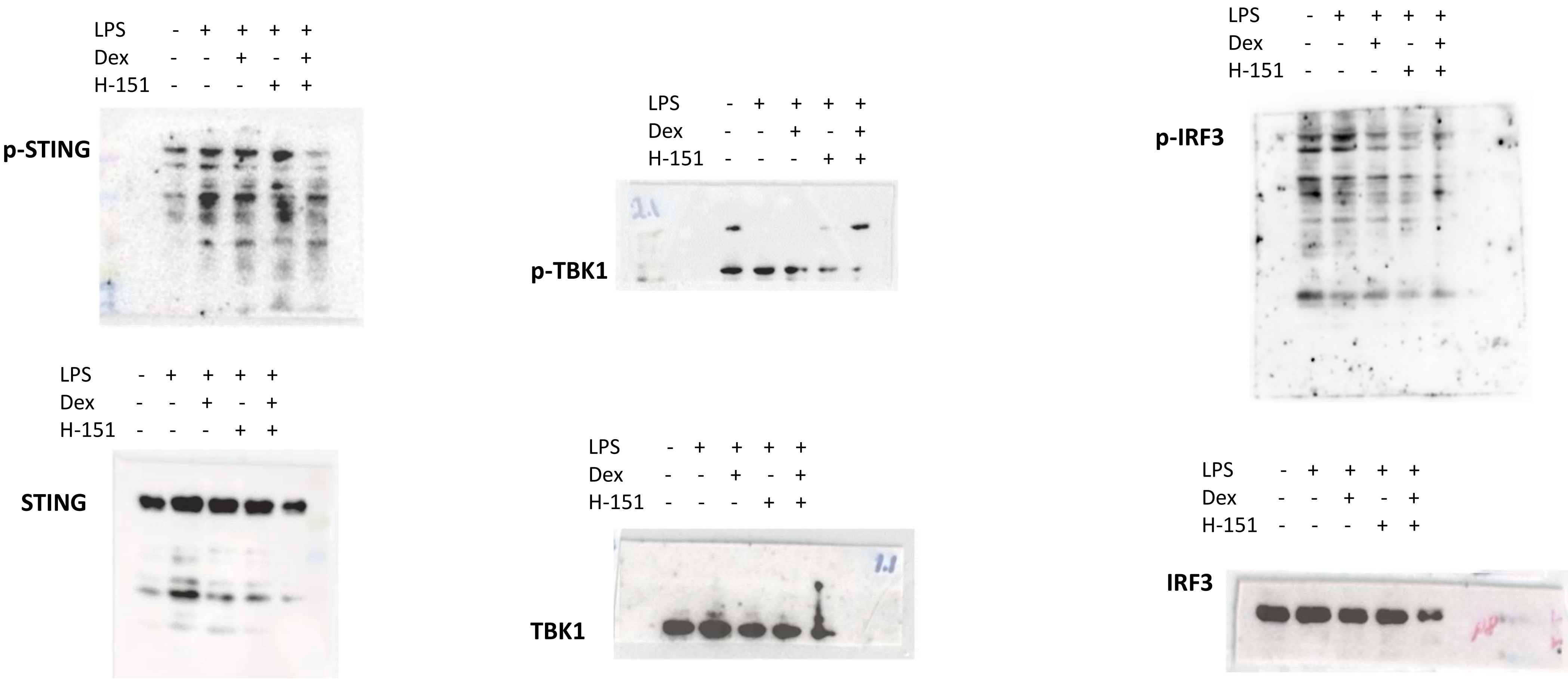

Figure D:

Smoker COPD human lung fibroblasts

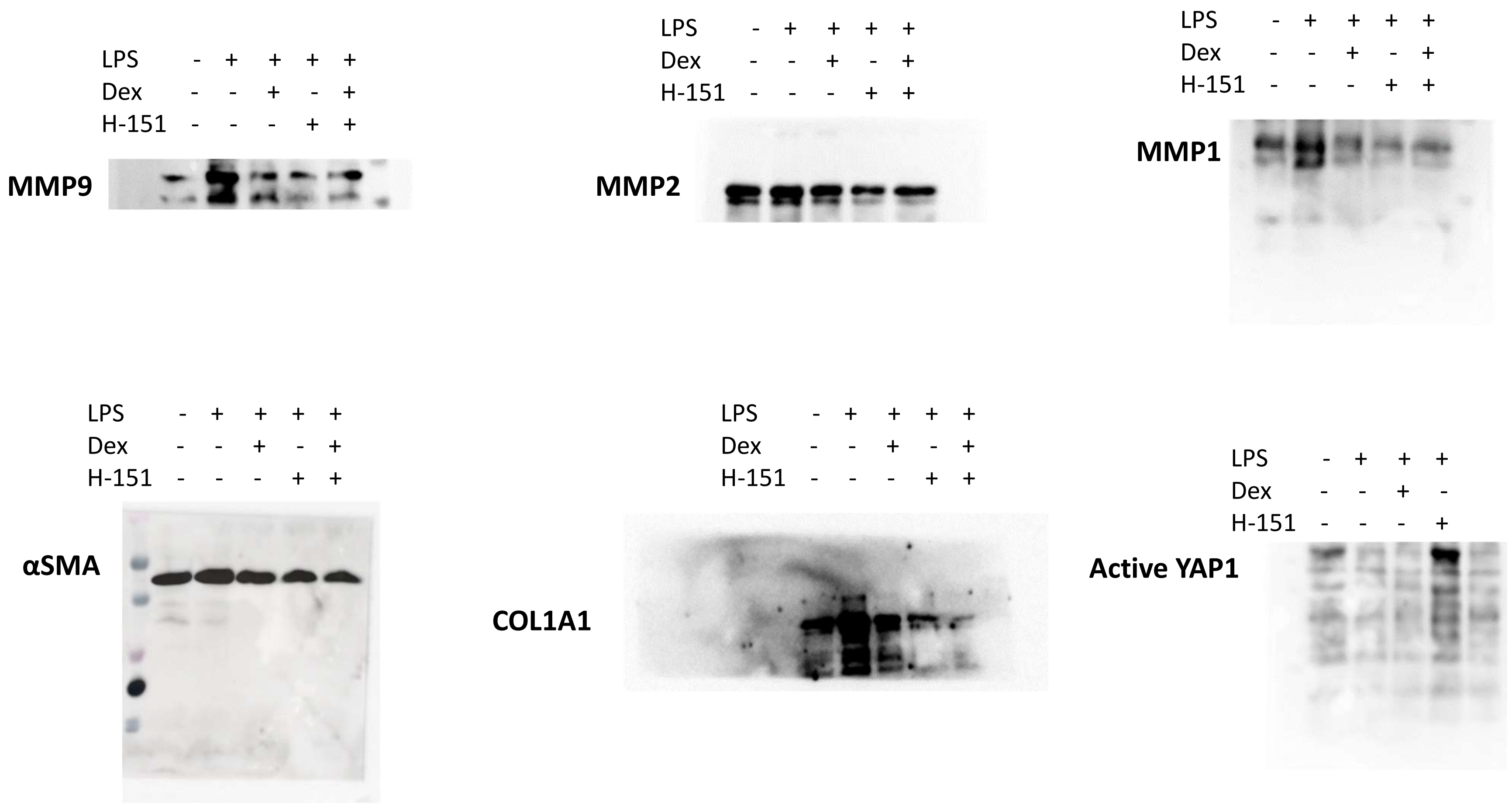

Supplement: S1 Raw images — (PDF) [file pone.0284061.s001.pdf]
